# Automatic coronavirus disease 2019 diagnosis based on chest radiography and deep learning – Success story or dataset bias?

**DOI:** 10.1002/mp.15419

**Published:** 2022-01-12

**Authors:** Jennifer Dhont, Cecile Wolfs, Frank Verhaegen

**Affiliations:** ^1^ Department of Radiation Oncology (Maastro) GROW School for Oncology Maastricht University Medical Centre+ Maastricht the Netherlands

**Keywords:** artificial intelligence, COVID‐19, dataset bias, X‐ray imaging

## Abstract

**Purpose:**

Over the last 2 years, the artificial intelligence (AI) community has presented several automatic screening tools for coronavirus disease 2019 (COVID‐19) based on chest radiography (CXR), with reported accuracies often well over 90%. However, it has been noted that many of these studies have likely suffered from dataset bias, leading to overly optimistic results. The purpose of this study was to thoroughly investigate to what extent biases have influenced the performance of a range of previously proposed and promising convolutional neural networks (CNNs), and to determine what performance can be expected with current CNNs on a realistic and unbiased dataset.

**Methods:**

Five CNNs for COVID‐19 positive/negative classification were implemented for evaluation, namely VGG19, ResNet50, InceptionV3, DenseNet201, and COVID‐Net. To perform both internal and cross‐dataset evaluations, four datasets were created. The first dataset Valencian Region Medical Image Bank (BIMCV) followed strict reverse transcriptase‐polymerase chain reaction (RT‐PCR) test criteria and was created from a single reliable open access databank, while the second dataset (COVIDxB8) was created through a combination of six online CXR repositories. The third and fourth datasets were created by combining the opposing classes from the BIMCV and COVIDxB8 datasets. To decrease inter‐dataset variability, a pre‐processing workflow of resizing, normalization, and histogram equalization were applied to all datasets. Classification performance was evaluated on unseen test sets using precision and recall. A qualitative sanity check was performed by evaluating saliency maps displaying the top 5%, 10%, and 20% most salient segments in the input CXRs, to evaluate whether the CNNs were using relevant information for decision making. In an additional experiment and to further investigate the origin of potential dataset bias, all pixel values outside the lungs were set to zero through automatic lung segmentation before training and testing.

**Results:**

When trained and evaluated on the single online source dataset (BIMCV), the performance of all CNNs is relatively low (precision: 0.65–0.72, recall: 0.59–0.71), but remains relatively consistent during external evaluation (precision: 0.58–0.82, recall: 0.57–0.72). On the contrary, when trained and internally evaluated on the combinatory datasets, all CNNs performed well across all metrics (precision: 0.94–1.00, recall: 0.77–1.00). However, when subsequently evaluated cross‐dataset, results dropped substantially (precision: 0.10–0.61, recall: 0.04–0.80). For all datasets, saliency maps revealed the CNNs rarely focus on areas inside the lungs for their decision‐making. However, even when setting all pixel values outside the lungs to zero, classification performance does not change and dataset bias remains.

**Conclusions:**

Results in this study confirm that when trained on a combinatory dataset, CNNs tend to learn the origin of the CXRs rather than the presence or absence of disease, a behavior known as short‐cut learning. The bias is shown to originate from differences in overall pixel values rather than embedded text or symbols, despite consistent image pre‐processing. When trained on a reliable, and realistic single‐source dataset in which non‐lung pixels have been masked, CNNs currently show limited sensitivity (<70%) for COVID‐19 infection in CXR, questioning their use as a reliable automatic screening tool.

## 1 INTRODUCTION

While vaccination programs are being rolled out, coronavirus disease 2019 (COVID‐19) maintains a strong grip on society worldwide.^1^ To limit the infection rate and avoid overburdening health care facilities, fast and effective screening and diagnosis remain critical in the fight against the severe acute respiratory syndrome coronavirus‐2 (SARS‐CoV‐2).^2^

Next to reverse transcriptase‐polymerase chain reaction (RT‐PCR) testing ‐ the current gold standard for diagnostic confirmation, both planar chest radiography (CXR) and computed tomography (CT) have been proposed as diagnostic solutions.^3^, ^4^, ^5^, ^6^, ^7^ Although the European Society of Radiology and European Society of Thoracic Imaging strongly advised against the use of CXR as a first‐line diagnostic technique, several early studies found that patients do present with abnormalities in CXR characteristic of COVID‐19.^8^, ^9^, ^10^ Together with the other benefits of this imaging modality, that is, relatively low cost and radiation dose, wide availability, speed and portability, these studies have led to the suggestion that CXR might be an ideal candidate for triaging patients presenting to hospitals, especially in epidemic areas.^11^

CXR for COVID‐19 diagnosis however still requires expert radiologists (>10 years of experience) to interpret the images with high specificity, a bottleneck in the workflow that is both time consuming and costly.^12^, ^13^ To overcome this issue, the artificial intelligence (AI) community has presented numerous machine – and deep learning (DL)‐based image analysis tools that are able to automatically differentiate between COVID‐19 positive and negative patients based on a single CXR, with reported accuracies and sensitivities often well over 90%.^14^, ^15^, ^16^, ^17^, ^18^, ^19^, ^20^, ^21^, ^22^, ^23^, ^24^, ^25^, ^26^, ^27^, ^28^, ^29^, ^30^, ^31^, ^32^, ^33^ One of the first of such networks was COVID‐Net, reaching 93.3% accuracy on the test set of their publicly available dataset termed COVIDx.^34^

As large single hospital CXR datasets of both COVID‐19 positive and negative patients are scarce, researchers looking into these DL methods have often made use of a combination of publicly available repositories.^35^, ^36^, ^37^ However, this approach can increase the risk of hidden biases that may lead to overly optimistic results.^38^, ^39^, ^40^, ^41^, ^42^, ^43^, ^44^ The likelihood of such a bias is particularly high when the data per class originates from different sources, such as different countries, hospitals, or imaging systems.^45^, ^46^ In these cases, underlying differences in the image data distributions, due to for example a difference in image acquisition parameters, post‐processing operations, or overall patient characteristics unrelated to COVID‐19, might create spurious correlations. Especially when these differences are more obvious than the COVID‐19 disease features, they are likely to be exploited by the neural network (NN). This phenomenon is known as short‐cut learning and hampers the NNs’ generalization capabilities significantly.^47^, ^48^

Jabbour et al. for example showed that NNs can accurately identify patient attributes in CXRs such as sex and age, and the NNs tend to exploit correlations between these attributes and the outcome label when learning to predict a diagnosis, leading to poor performance when such correlations do not hold in the test population.^46^ In a study by Kim et al., underlying differences in dataset distributions of commonly used COVID‐19 CXR datasets were visualized through principal component analysis and t‐distributed stochastic neighbor embedding.^48^ Excellent performance during internal validation and poor performance in external validation showed these differences were likely exploited by the NNs during training. In the same context but by using state‐of‐the‐art techniques in explainable AI, DeGrave et al. also showed that NNs are more likely to rely on confounding factors rather than relevant pathology.^47^

The aim of this study was to qualitatively and quantitatively investigate to what extent possible dataset bias has influenced the performance of a range of promising deep convolutional NNs (CNNs) that were previously proposed for COVID‐19 diagnosis in CXRs. In addition, through the creation of saliency maps and additional pre‐processing, we aimed to define what exactly caused the dataset bias in a widely used COVID‐19 CXR dataset, to support the development of bias elimination methods. Finally, we determined what performance can be expected with current CNN architectures on a reliable dataset that carries a low risk of dataset bias and is publicly available.

## 2 MATERIALS AND METHODS

Five deep CNNs that were previously proposed for automatic COVID‐19 positive/negative classification were implemented for evaluation: (1) VGG19,^17^, ^19^, ^49^ (2) ResNet50,^15^, ^50^ (3) InceptionV3,^51^, ^52^, ^53^ (4) DenseNet201^54^, ^55^, ^56^, and (5) COVID‐Net.^34^ As can be seen in Table S1, these CNNs cover a broad range of layers and number of trainable parameters, while also differing in topology (e.g., skip connections in ResNet50, parallel connections in InceptionV3, or multiple direct connections from previous layers in DenseNet201).

Of these five CNNs, only COVID‐Net was specifically designed for COVID‐19 detection on CXRs. COVID‐Net was developed as an open‐source initiative and several versions are publicly available including a binary (COVID‐19 positive/negative) and multi‐class (no pneumonia/non‐COVID‐19 pneumonia/COVID‐19 pneumonia) classification network.^57^ Pre‐trained models are available online, together with several scripts to pre‐process the corresponding dataset and train and test the network. In this study, COVID‐Net for binary classification was implemented as provided online without any modifications and the latest pre‐trained model weights (‘COVID‐Net‐CXR‐2′, released on 20 March 20 2021) were downloaded. CNNs 1–4 were applied as implemented in Keras using the Tensorflow backend.^58^, ^59^ All four networks were pre‐trained on ImageNet and further trained (all layers) on the datasets described in the next paragraphs. Hyper‐parameters were optimized for each dataset using 20% of the training sets as validation and early stopping based on the validation loss was applied. For reproducibility, a detailed overview of all hyper‐parameters is given in Table S1.

**FIGURE 1:**
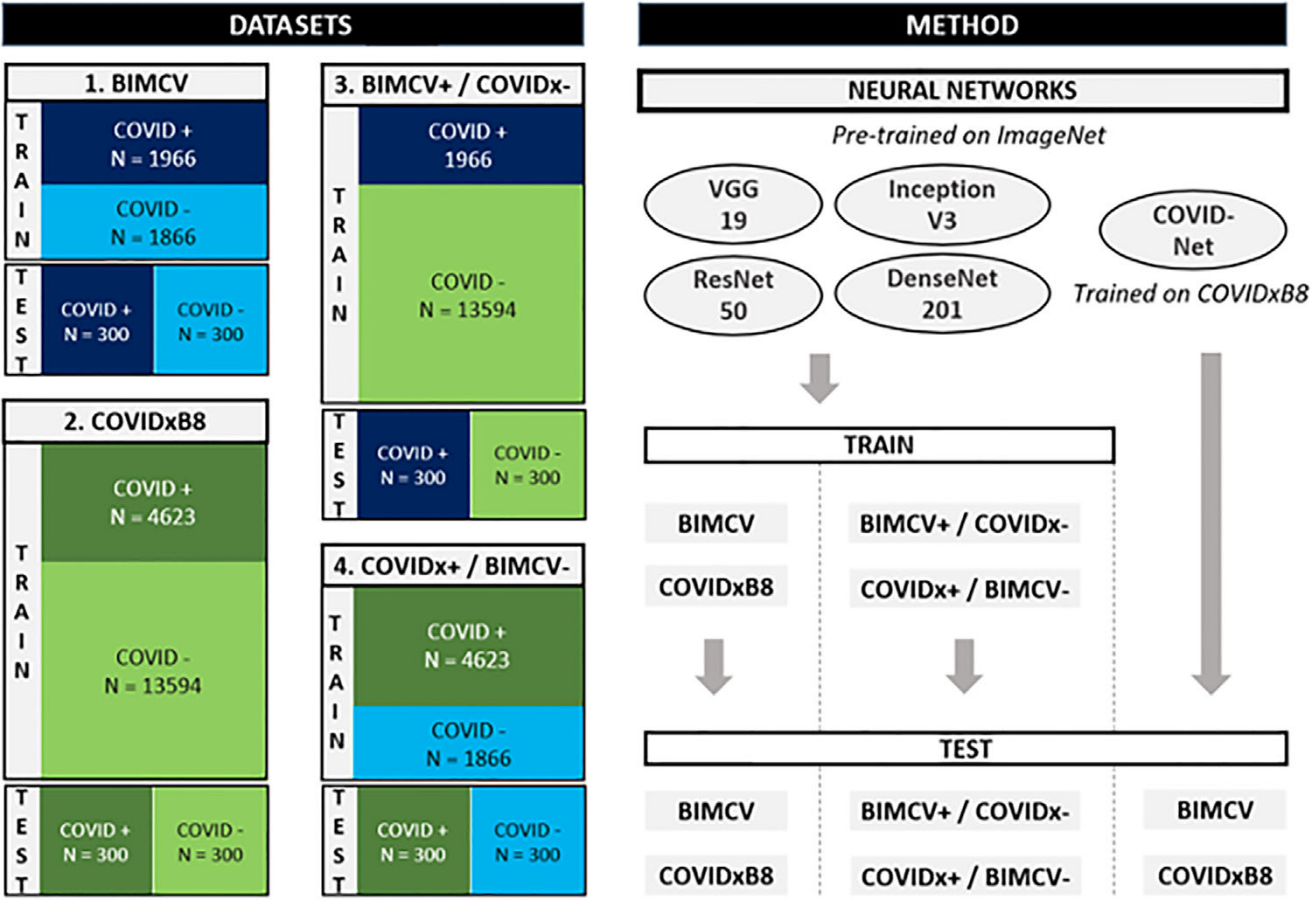
Illustration of the four different datasets with chest radiography (CXRs) of coronavirus disease 2019 (COVID‐19)‐positive (COVID +) and ‐negative (COVID–) patients, including the number of CXRs per class used for training and testing (left), together with a schematic overview of the methodology (right)

To perform both internal and cross‐dataset (i.e., mimicking external) evaluations to quantify generalizability and to evaluate the influence of multiple sources in a single dataset, four COVID‐19 positive/negative datasets were created as illustrated in Figure 1: (1) Valencian Region Medical Image Bank (BIMCV), (2) COVIDxB8, (3) BIMCV+/COVIDx–, and (4) COVIDx+/BIMCV–. The original datasets from which BIMCV and COVIDxB8 were created represent two of the largest publicly available datasets of COVID‐19 medical images and are therefore often used in studies investigating DL for automatic COVID‐19 diagnosis.

BIMCV was created from a single online source of CXRs, namely the Medical Imaging Databank in the BIMCV‐COVID‐19 dataset which contains both CXRs as well as CT data.^35^ CXRs in this dataset originate from 11 medical centers in the Valencian region (Spain) and were acquired in the period between 26 February 26 and 18 April 2020. To ensure reliable labeling, only those CXRs that could be linked to a positive or negative RT‐PCR test performed on the same day, as reported in the accompanying metadata, were included. Of note is that the COVID‐19 negative class contains both normal CXRs as well as CXRs of confirmed bacterial or non‐COVID‐19 viral pneumonia. Of the BIMCV dataset, 300 COVID‐19 positive and 300 negative CXRs (randomly selected) were set aside for testing, ensuring all images per patient belonged to a single set.

COVIDxB8 is the latest version of COVIDx; a publicly available dataset created specifically for the development of COVID‐Net.^34^ COVIDx is an open‐access benchmark dataset created through the combination and modification of six online open access data repositories containing CXRs of varying sources. Of note is that all COVID‐19 negative images originate from the RSNA Pneumonia Detection Challenge, including both normal, bacterial, and non‐COVID‐19 viral pneumonia, while the COVID‐19 positive images are collected from five other online repositories.^37^ In the latter, both the origin and how the ground truth label was established are unspecified for most CXRs. Using the dataset creation and pre‐processing scripts provided on the COVID‐Net project GitHub page, the latest version of the COVIDx binary dataset was created (both training and test set).^57^ To avoid class imbalance and to increase the test set size, 200 COVID‐19 negative and 26 positive CXRs were randomly selected from the training set and added to the test set, resulting in 300 CXRs per class.

The third (BIMCV+/COVIDx–) and fourth (COVIDx+/BIMCV–) datasets with mixed sources were created by combining the opposing classes from the BIMCV and COVIDxB8 datasets, see Figure 1. Corresponding test sets were created by combining the respective test set classes.

Both BIMCV and COVIDxB8 contain CXRs in non‐medical image formats (.png and .jpg), different sizes and different data types (uint8 and uint16). To decrease inter‐dataset variability, all CXRs were pre‐processed by resizing to 512 × 512 pixels (smallest image size present in the original datasets), normalization between 0 and 1, histogram equalization, and conversion to uint8.^60^ Images were not converted from RGB to grayscale as all CNNs require 3‐channel input. Figure S1 illustrates the different datasets after pre‐processing.

Evaluation of the classification performance of each CNN was performed internally (i.e., test set with the same origin as the training set) and cross‐dataset (i.e., test set with different origin as the training set), as illustrated in Figure 1. To quantify the classification performance, precision (= positive predictive value) and recall (= sensitivity) at a fixed classification threshold of 50% probability were calculated. Further, as high sensitivity for COVID‐19 infection is desirable in a screening scenario, precision at 90% recall was also determined for each dataset for CNNs 1–4.

Lastly, to increase the understanding of the CNNs decision‐making, saliency maps that link the CNN classification outcome to the areas in the input image that had the most impact on that outcome were created.^61^, ^62^, ^63^, ^64^ Through this attribution method, a qualitative sanity check can be performed by evaluating whether the high‐impact areas correspond to relevant areas inside the lung as opposed to improper information in the images (e.g., areas outside the lung, embedded symbols, etc.). XRAI, a region‐based attribution method based on Integrated Gradients was applied and saliency maps showing the most salient segments (top 5%, 10%, and 20%) of each CXR were visualized and qualitatively evaluated.^65^, ^66^ Further, in an additional experiment, the pixel values outside the lungs were set to zero through automatic lung segmentation, using a U‐Net CNN, on all CXRs before training and testing. As such, the CNNs were forced to use only relevant parts of the anatomy and could not rely on embedded text or symbols which are typically present outside the lungs. This is to further investigate the source of possible dataset bias and a potential solution.

## 3 RESULTS

Table 1 displays the performance of each CNN for all datasets and both the internal and cross‐dataset evaluations. When trained and tested on BIMCV, precision, and recall of all the CNNs are relatively low, ranging from 0.65 to 0.72 and from 0.59 to 0.71, respectively. This performance varies slightly in the cross‐dataset evaluation on the test set of COVIDxB8, with precision and recall ranging from 0.58 to 0.82 and from 0.57 to 0.72, respectively. When trained and tested on COVIDxB8, all CNNs reached the highest precision (≥0.96) and relatively high sensitivity (range: 0.77–0.85). However, when subsequently evaluated cross‐dataset on the test set of BIMCV, precision, and recall of all the CNNs decreased substantially ranging from 0.55 to 0.61 and from 0.41 to 0.55, respectively.

**TABLE 1:** Coronavirus disease 2019 (COVID‐19) positive precision/recall obtained on the unseen test set of each dataset in both the internal (grey shading) and cross‐dataset evaluation. BIMCV+/COVIDx– and COVIDx+/BIMCV– were created by combining the opposing classes from the Valencian Region Medical Image Bank (BIMCV) and COVIDxB8 datasets. Numbers in bold indicate the particularly poor performance when the origin of the two classes is reversed between training and testing

	**Training set**	**Test set** **BIMCV**	**COVIDxB8**	**BIMCV+ /COVIDx**–	**COVIDx+ /BIMCV**–
1. VGG19	BIMCV	0.72/0.71	0.58/0.72	–	–
	COVIDxB8	0.56/0.44	0.98/0.85	–	–
	BIMCV+/COVIDx‐	–	–	0.98/0.99	**0.30/0.42**
	COVIDx+/BIMCV‐	–	–	**0.10/0.05**	1.00/0.94
2. ResNet50	BIMCV	0.74/0.66	0.58/0.62	–	–
	COVIDxB8	0.61/0.48	0.98/0.77	–	–
	BIMCV+/COVIDx‐	–	–	0.96/0.98	**0.38/0.58**
	COVIDx+/BIMCV‐	–	–	**0.32/0.08**	0.98/0.85
3. InceptionV3	BIMCV	0.65/0.65	0.63/0.63	–	–
	COVIDxB8	0.60/0.55	0.98/0.84	–	–
	BIMCV+/COVIDx‐	–	–	0.95/0.98	**0.40/0.65**
	COVIDx+/BIMCV‐	–	–	**0.16/0.04**	0.99/0.84
4. DenseNet201	BIMCV	0.76/0.59	0.82/0.57	–	–
	COVIDxB8	0.58/0.55	0.96/0.87	–	–
	BIMCV+/COVIDx‐	–	–	0.94/1.00	**0.44/0.80**
	COVIDx+/BIMCV‐	–	–	**0.13/0.04**	1.00/0.84
5. COVID‐Net	COVIDxB8	0.55/0.41	0.99/0.77	–	–

Similar results to those obtained with COVIDxB8 were obtained for all CNNs when trained on one of the other combinatory datasets (e.g., BIMCV+/COVIDx– and COVIDx+/BIMCV–). Moreover, it can be observed that when a CNN is trained on BIMCV+/COVIDx– and evaluated cross‐dataset on COVIDx+/BIMCV– (or vice versa), that is, when the origin of the two classes is switched between training and testing, CNNs perform worse than if classification would have occurred at random, with sensitivities ranging only from 0.04 to 0.08 (indicated in bold in Table 1). These results strongly suggest that when trained on a dataset in which the classes originate from different (online) sources, the CNNs learn confounding factors related to the data source rather than medically relevant pathology in the CXRs.

Table S2 lists the classification precision that will be obtained with CNNs 1–4 when 90% sensitivity for COVID‐19 positive detection is required. A similar trend in results as with the previous metrics can be observed across the different datasets and for all CNNs. Large discrepancies exist between the internal and cross‐dataset evaluations when CNNs are trained on a combinatory dataset, while more consistent results are obtained when trained on BIMCV. However, as precision values range between 0.51 and 0.58 in the cross‐dataset evaluation, the automatic screening performance currently seems below clinical utility.

**FIGURE 2:**
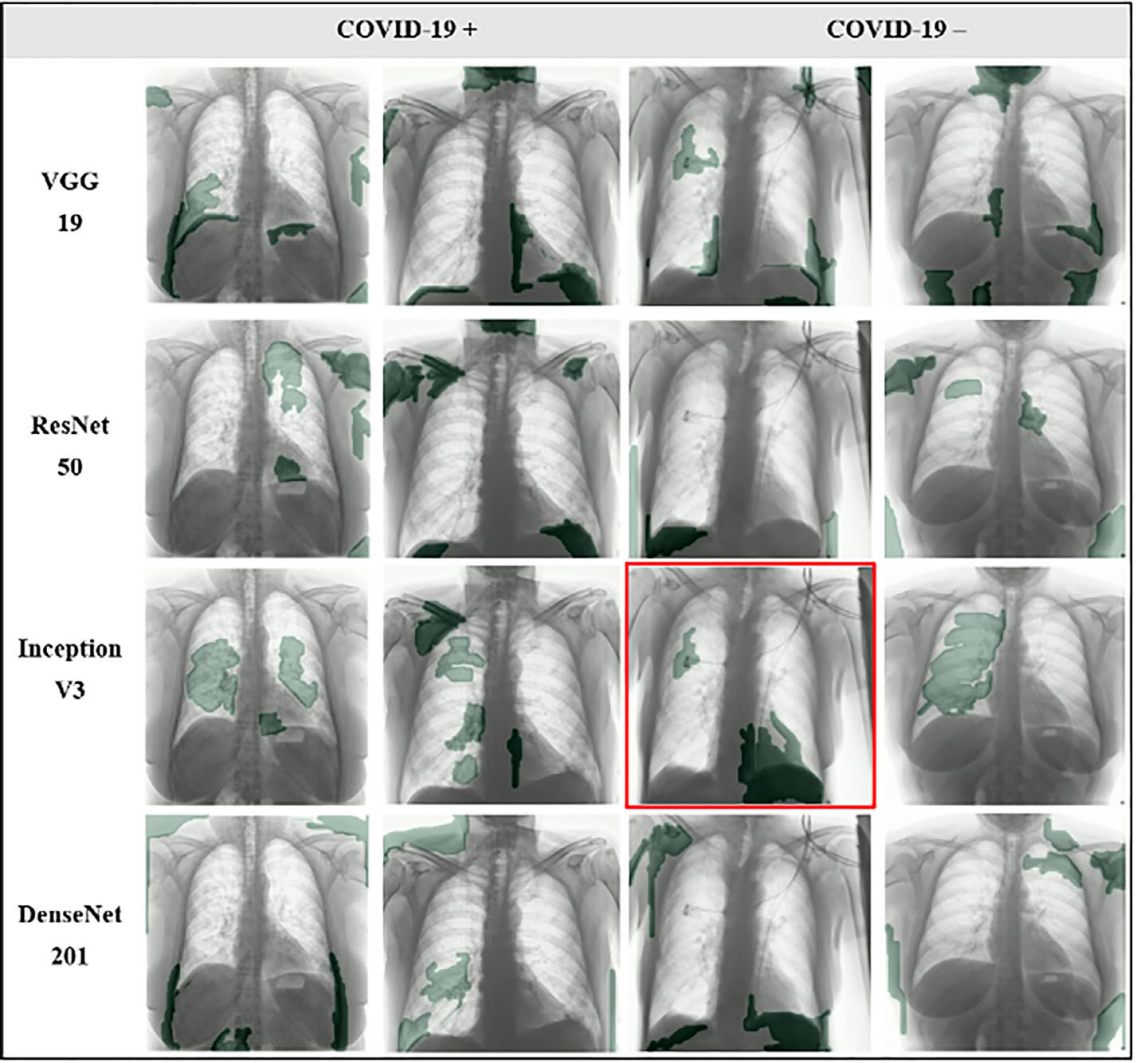
Four representative examples (two coronavirus disease 2019 [COVID‐19] positive, two COVID‐19 negative) of the saliency maps obtained for convolutional neural network (CNN) 1–4 trained on the Valencian Region Medical Image Bank (BIMCV) dataset, showing the most salient segments (top 10%, in green). All images originate from the BIMCV test set. Chest radiography (CXRs) delineated in red were misclassified

Next to the quantitative results, a qualitative sanity check was performed by visualizing saliency maps that link the CNNs classification outcome to the most impactful segments in the input CXR. Figures 2 (BIMCV) and 3 (COVIDxB8) visualize the top 10% most salient segments for four representative examples from the internal test sets. Other examples of the top 5% and 20% most salient segments can be found in Figures S2–S5.

**FIGURE 3:**
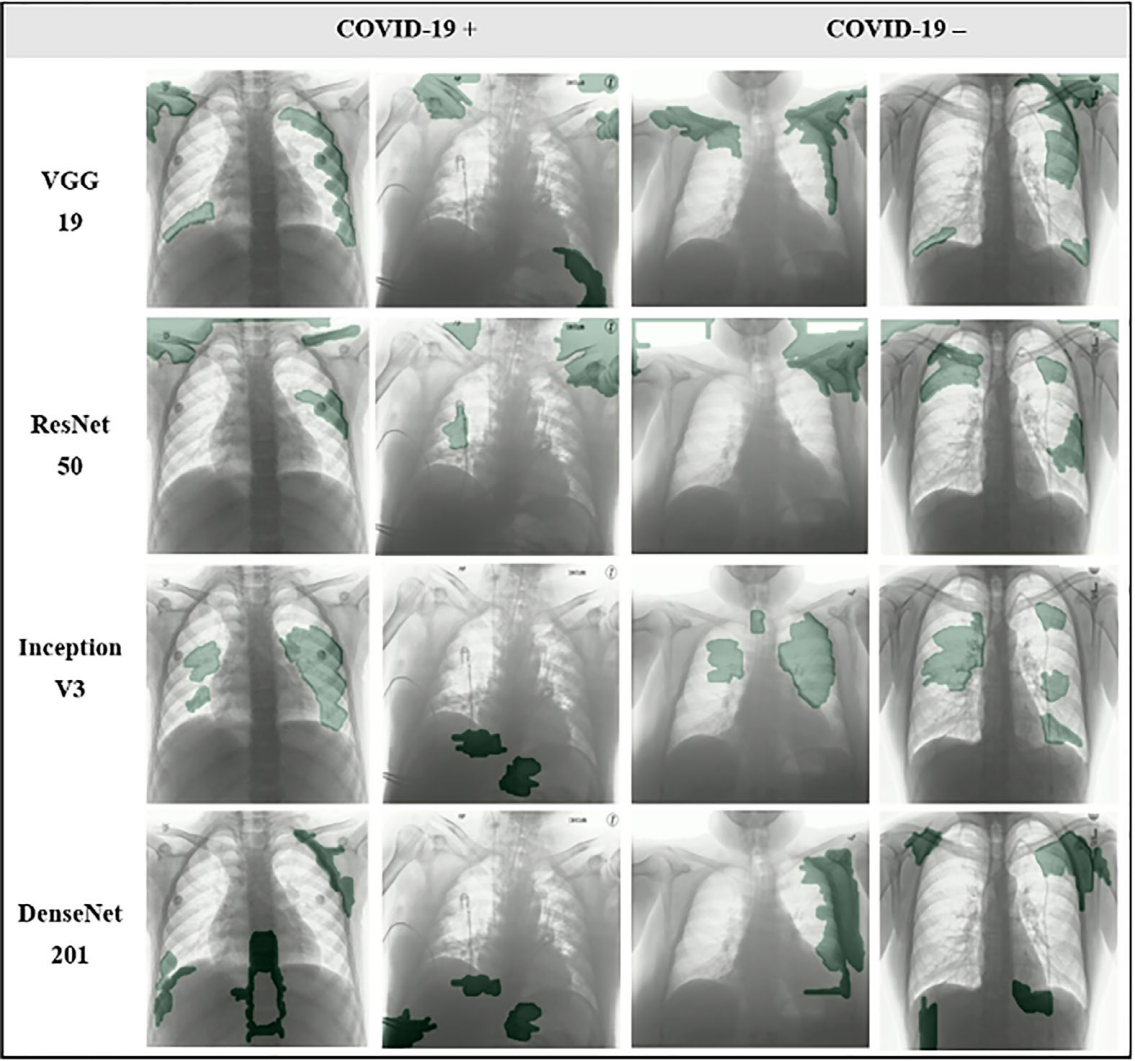
Four representative examples (two coronavirus disease 2019 [COVID‐19] positive and two COVID‐19 negative) of the saliency maps obtained for convolutional neural network (CNN) 1–4 trained on the COVIDxB8 dataset, showing the most salient segments (top 10%, in green). All images originate from the COVIDxB8 test set and were correctly classified by each network

For both BIMCV and COVIDxB8, it can be observed that often the most impactful regions used for decision making do not correspond with COVID‐19 lesions and are frequently located outside the lungs. Further, areas, where embedded text and/or symbols can be present, are often part of the top salient segments. Figures 2 and 3 also show that despite similar numerical results, large discrepancies exist in the most salient segments between the different CNNs.

When CXR pixels outside the lungs are masked before training and testing, the classification performance of CNNs 1–4 when trained on COVIDxB8 does not change (Table 2 vs. Table 1). This indicates the dataset bias remains and is due to differences in overall intensities (e.g., contrast, noise, etc.), rather than embedded symbols or text. For BIMCV and all evaluated CNNs, masking irrelevant parts of the CXRs led to more consistent results between internal and cross‐dataset evaluation, but performance remains relatively low (cross‐dataset sensitivity <0.70).

**TABLE 2:** Coronavirus disease 2019 (COVID‐19) positive precision/recall obtained on the unseen test set of each dataset, in both the internal (grey shading) and cross‐dataset evaluation, when pixels outside the lungs are masked before training and testing

		**Test set**	
	**Training set**	**BIMCV**	**COVIDxB8**
1. VGG19	BIMCV	0.68/0.65	0.68/0.69
	COVIDxB8	0.61/0.43	0.97/0.80
2. ResNet50	BIMCV	0.67/0.71	0.67/0.64
	COVIDxB8	0.63/0.64	0.97/0.87
3. InceptionV3	BIMCV	0.65 / 0.64	0.66/0.60
	COVIDxB8	0.61/0.51	0.98/0.80
4. DenseNet201	BIMCV	0.69/0.56	0.82/0.57
	COVIDxB8	0.63/0.71	0.95/0.86

Abbreviation: BIMCV, Valencian Region Medical Image Bank.

## 4 DISCUSSION

The evaluation of five distinct CNNs that were previously proposed for automatic COVID‐19 diagnosis on CXR showed quantitative results that were highly dependent on the applied dataset (Table 1). Moreover, all CNNs failed a qualitative sanity check on all datasets, despite consistent performance between internal and external evaluation when trained on the single‐source dataset (Figures 2 and 3).

The five CNNs evaluated in this study were selected to represent a broad range of trainable parameters, number of layers, and topologies (Table S1). However, before discussing the results it should be noted that there is no certainty the results obtained with these models are representative of all NN architectures. Similarly, the two datasets selected for this study, while representing some of the most used publicly available datasets on the topic, might not be representative for all datasets.

Quantitatively, all CNNs showed similar performance (Table 1). However, an extensive evaluation of COVID‐Net is limited as only pre‐trained models are available. While the network performs well when trained and tested on the COVIDxB8 dataset, the quality of the latter is questionable. Large discrepancies between internal and cross‐dataset evaluations, seen with each of the five CNNs, indicate the CNNs are able to learn other patterns in the dataset that distinguishes the two classes, but that is not related to the presence of COVID‐19 infection. These results persist even when pixels outside the lungs are masked before training and testing, despite an identical pre‐processing workflow in which the image intensities are normalized and spread out homogeneously over a fixed intensity interval through histogram equalization.

Tartaglione et al. previously warned for possible hidden bias when combining different datasets, noting that NNs might find spurious correlations in different imaging parameters between datasets instead of looking at the actual disease.^40^ The latter was also confirmed by Maguolo and Nanni, who showed deep NNs could still identify the origin of the CXRs while the lung regions were excluded from the images.^67^ Of interest, the dataset of CXRs used by Maguolo and Nanni is included in COVIDxB8. The presence of a hidden bias is further confirmed by the cross‐dataset evaluation of datasets 3 and 4 in this study, showing that the CNNs continue to classify images according to the dataset they belong to, instead of the presence or absence of disease (Table 1, numbers in bold). A qualitative sanity check through the use of saliency maps also confirms the CNNs decision‐making is largely based on regions outside the lung, including but not limited to embedded text and/or symbols, instead of COVID‐19 lesions or healthy lung tissue (Figures 2 and 3).

Although the authors of COVID‐Net have also used an explainability approach and their qualitative results indicated COVID‐Net often used relevant areas in the CXR for decision making, results in the current study indicate a quantitative external validation remains crucial.^68^, ^69^ It is therefore recommended to limit the use of COVIDx and other combinatory datasets in their current form ‐ pending novel pre‐processing techniques that are able to robustly eliminate dataset bias and to interpret the results of models trained on such datasets with care.^70^

By creating a relatively large dataset from a single online source, the aim of the BIMCV dataset was to eliminate this bias and obtain more realistic results. Further, by adhering to a strict RT‐PCR ground‐truth for each CXR, a dataset with highly reliable labels was created. However, it has to be taken into account that the RT‐PCR test has high specificity but a moderate sensitivity rate, and so an unknown percentage of false negatives might still be present in the final dataset.^71^, ^72^ The latter represents an almost unavoidable obstacle in the (semi‐)automatic creation of very large COVID‐19 datasets required for DL unless a reliable amount of additional and structured metadata is available on the patient's symptoms and follow‐up tests.^73^

Further, by adhering only to RT‐PCR criteria, the BIMCV dataset likely contains a percentage of mild COVID‐19 positive cases with limited symptoms and no radiological signs.^74^ This might partly explain the lower COVID‐19 classification performance obtained in this study on BIMCV compared to similar studies on other datasets. However, we believe BIMCV represents a clinically realistic scenario when applying CXR for screening and diagnosis, as not all patients will present with severe COVID‐19 pneumonia. This however also implies that automatic COVID‐19 diagnosis using CXR and DL has limited sensitivity (range: 0.59–0.71), in combination with low specificity (range: 0.56–0.76). Furthermore, a qualitative sanity check revealed the NNs do not focus on relevant information in the CXRs. These results indicate that a quantitative external validation alone might not be sufficient to ensure a NN relies on medically relevant pathology, as also concluded by DeGrave et al.^47^ By segmenting the lung regions as an additional pre‐processing step before feeding the CXRs to the classification networks, CNNs were forced to look at relevant parts of the anatomy only and generalizability improved slightly. However, COVID‐19 sensitivity and precision remained below 70%. As the BIMCV dataset is publicly available, the pre‐processing steps mentioned in this study, including lung segmentation, can be followed to create a relatively large and reliable dataset with a low risk of bias for further CNN development.

Improvements can be expected through a number of approaches such as the optimization of NN architectures and/or the incorporation of clinical patient features such as COVID‐19 specific symptoms in the final NN decision making.^75^, ^76^ Additional improvements can be expected from the availability of more standardized, large‐scale, and qualitative datasets, provided in medical image standards such as DICOM so differences in overall intensity values (e.g., contrast, noise, etc.) can be eliminated. In addition, novel data augmentation techniques such as those using generative adversarial networks to simulate pathology in existing CXRs or render completely synthetic CXRs could create larger and more balanced datasets.^77^, ^78^, ^79^ Another approach is presented by Ahmed et al., who propose fine‐tuning on unseen data to improve the performance at a new site.^80^

## 5 CONCLUSIONS

Over the last 2 years, the AI community has presented several automatic screening tools for COVID‐19 based on CXR, with reported accuracies often well over 90%. However, it has been noted that many of these studies have likely suffered from dataset bias, leading to overly optimistic results. This study confirms that when trained on a combinatory dataset, CNNs tend to learn the origin of the CXRs rather than the presence or absence of disease, a behavior known as short‐cut learning. The bias is shown to originate from differences in overall pixel values rather than embedded text or symbols, despite consistent image pre‐processing. When trained on a reliable, and realistic single‐source dataset in which non‐lung pixels have been masked, CNNs currently show limited sensitivity (<70%) for COVID‐19 infection in CXR.

## CONFLICT OF INTEREST

The authors declare that they have no conflict of interest.

BIMCV‐COVID19: https://bimcv.cipf.es/bimcv‐projects/bimcv‐covid19/

COVIDx: https://github.com/lindawangg/COVID‐Net/blob/master/docs/COVIDx.md

## Supporting information

Supporting Information

## Data Availability

The data that support the findings of this study are available in the following repositories in the public domain:
