# Supplementary material for: Automatic coronavirus disease 2019 diagnosis based on chest radiography and deep learning – Success story or dataset bias?
Source: Med Phys. 2022 Jan 12;49(2):978–87. doi: 10.1002/mp.15419 (PMC9015341; doi:10.1002/mp.15419)
Supplement: Supplementary file 1 — Supporting Information [file MP-49--s001.doc]

**Supplementary Figure Legends**


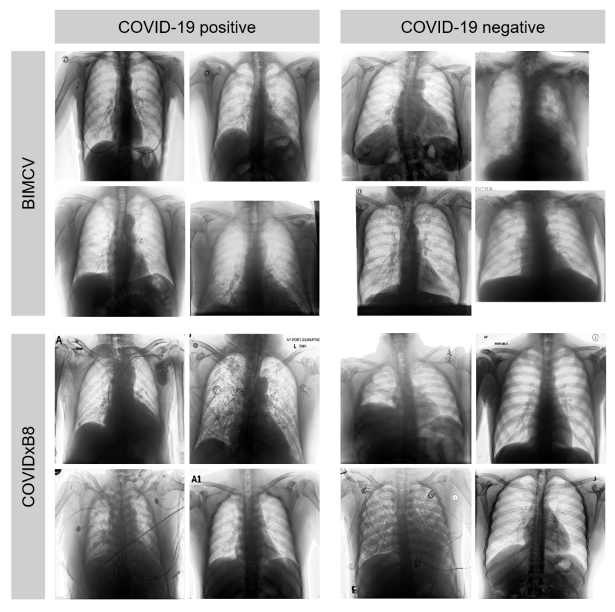


**Figure S1**: Example of CXRs from the COVID-19 positive (left) and negative (right) class of the BIMCV (top) and COVIDxB8 (bottom)› datasets.


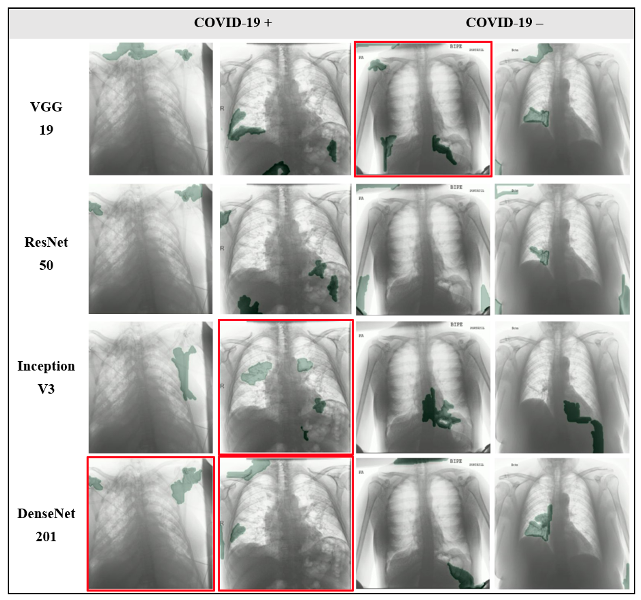


**Figure S2:** Four representative examples (two COVID-19 positive, two COVID-19 negative) of the saliency maps obtained for CNN 1 to 4 trained on the BIMCV dataset, showing the most salient segments (top 5%). All images originate from the BIMCV test set. CXRs delineated in red were misclassified.


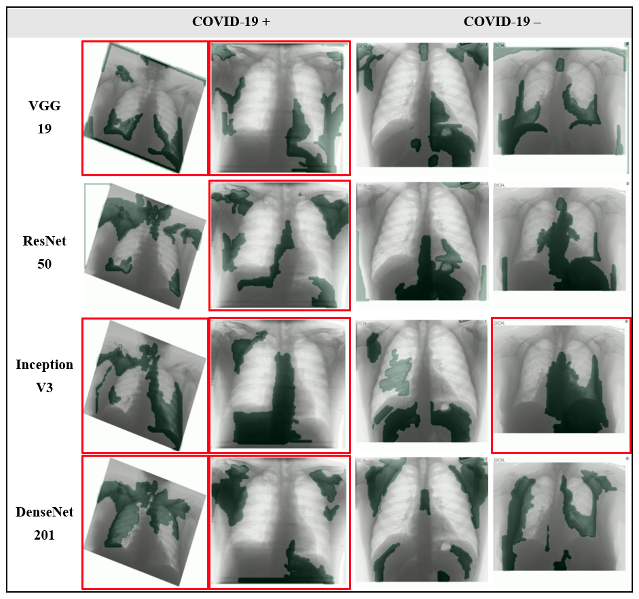


**Figure S3:** Four representative examples (two COVID-19 positive, two COVID-19 negative) of the saliency maps obtained for CNN 1 to 4 trained on the BIMCV dataset, showing the most salient segments (top 20%). All images originate from the BIMCV test set. CXRs delineated in red were misclassified.


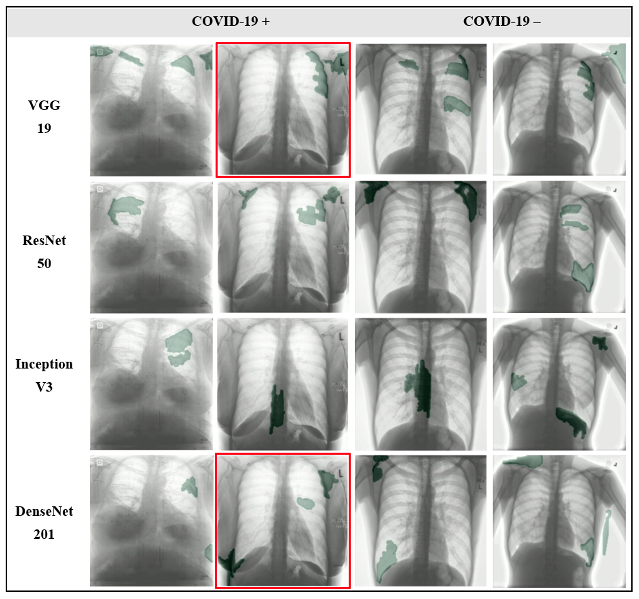


**Figure S4:** Four representative examples (two COVID-19 positive, two COVID-19 negative) of the saliency maps obtained for CNN 1 to 4 trained on the COVIDxB8 dataset, showing the most salient segments (top 20%). All images originate from the COVIDxB8 test set. CXRs delineated in red were misclassified.


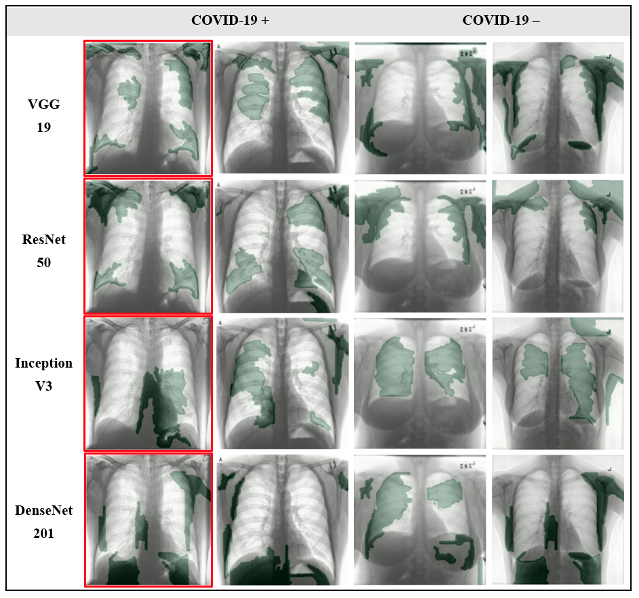


**Figure S5:** Four representative examples (two COVID-19 positive, two COVID-19 negative) of the saliency maps obtained for CNN 1 to 4 trained on the COVIDxB8 dataset, showing the most salient segments (top 20%). All images originate from the COVIDxB8 test set. CXRs delineated in red were misclassified.

**Table S1:** Overview of hyper-parameters for neural networks VGG19, ResNet50, InceptionV3 and DenseNet201 applied for all datasets.

|  | **VGG19** | **ResNet50** | **InceptionV3** | **DenseNet201** | **COVID-Net** |
| --- | --- | --- | --- | --- | --- |
| Number of layers | 26 | 50 | 159 | 201 | 82 |
| Number of trainable parameters | 143.67 ∙ 10^6^ | 25.64 ∙ 10^6^ | 23.85 ∙ 10^6^ | 20.24 ∙ 10^6^ | 11.75 ∙ 10^6^ |
| Image input size (pixels) | 512 x 512 x 3 | 512 x 512 x 3 | 512 x 512 x 3 | 512 x 512 x 3 | 480 x 480 x 3 |
| Top classifier dense layer |  |  |  |  |  |
| Number of nodes | 1024 | 1024 | 1024 | 1024 |  |
| Activation function | Rectified Linear Unit | Rectified Linear Unit | Rectified Linear Unit | Rectified Linear Unit |  |
| Top classifier last layer |  |  |  |  |  |
| Activation function | Softmax | Softmax | Softmax | Softmax |  |
| Initial learning rate | 10^-3^ | 10^-5^ | 10^-5^ | 10^-5^ |  |
| Batch size | 16 | 8 | 8 | 4 | 20 |
| Loss | categorical cross entropy | categorical cross entropy | categorical cross entropy | categorical cross entropy |  |
| Optimizer | Adam | Adam | Adam | Adam |  |
| β1 | 0.9 | 0.9 | 0.9 | 0.9 |  |
| β2 | 0.999 | 0.999 | 0.999 | 0.999 |  |
| epsilon | 0.05 | 0.01 | 0.001 | 0.01 |  |
| Early stopping | yes | yes | yes | yes |  |
| based on | validation loss | validation loss | validation loss | validation loss |  |
| patience | 5 epochs | 5 epochs | 5 epochs | 5 epochs |  |

**Table S2:** COVID-19 positive precision at 90% recall obtained on the test set of each dataset in both the internal (grey shading) and cross-dataset evaluation. Numbers in bold indicate the particularly poor performance when the data set is switched between classes from training to testing.

|  |  | ***Test set*** | | | |
| --- | --- | --- | --- | --- | --- |
|  | ***Training set*** | ***BIMCV*** | ***COVIDxB8*** | ***BIMCV+/***  ***COVIDx-*** | ***COVIDx+/***  ***BIMCV-*** |
| VGG19 | ***BIMCV*** | 0.60 | 0.53 | - | - |
|  | ***COVIDxB8*** | 0.50 | 0.97 | - | - |
|  | ***BIMCV+/COVIDx-*** | - | - | 1.00 | **0.47** |
|  | ***COVIDx+/BIMCV-*** | - | - | **0.48** | 1.00 |
| ResNet50 | ***BIMCV*** | 0.58 | 0.51 | - | - |
|  | ***COVIDxB8*** | 0.51 | 0.96 | - | - |
|  | ***BIMCV+/COVIDx-*** | - | - | 0.99 | **0.47** |
|  | ***COVIDx+/BIMCV-*** | - | - | **0.50** | 0.95 |
| InceptionV3 | ***BIMCV*** | 0.54 | 0.54 | - | - |
|  | ***COVIDxB8*** | 0.52 | 0.95 | - | - |
|  | ***BIMCV+/COVIDx-*** | - | - | 0.99 | **0.47** |
|  | ***COVIDx+/BIMCV-*** | - | - | **0.49** | 0.97 |
| DenseNet201 | ***BIMCV*** | 0.61 | 0.58 | - | - |
|  | ***COVIDxB8*** | 0.51 | 0.96 | - | - |
|  | ***BIMCV+/COVIDx-*** | - | - | 1.00 | 0.47 |
|  | ***COVIDx+/BIMCV-*** | - | - | 0.50 | 0.97 |
